# Supplementary material for: Association of P10L Polymorphism in Melanopsin Gene with Chronic Insomnia in Mexicans
Source: Int J Environ Res Public Health. 2021 Jan 12;18(2):571. doi: 10.3390/ijerph18020571 (PMC7827055; doi:10.3390/ijerph18020571)
Supplement: Supplementary file 1 [file ijerph-18-00571-s001.pdf]

Supplementary Material

```
  1 MNPPSGPRVPPSPTQEPSCMATPAPPSWWDSSQSSISSLGRLPSISPTAPGTWAAAWVPL    60
 61 PTVDVPDHAHYTLGTVILLVGLTGMLGNLTVIYTFCRSRSLRTPANMFIINLAVSDFLMS   120
121 FTQAPVFFTSSLYKQWLFGETGCEFYAFCGALFGISSMITLTAIALDRYLVITRPLATFG   180
181 VASKRRAAFVLLGVWLYALAWSLPPFFGWSAYVPEGLLTSCSWDYMSFTPAVRAYTMLLC   240
241 CFVFFLPLLIIIYCYIFIFRAIRETGRALQTFGACKGNGESLWQRQRLQSECKMAKIMLL   300
301 VILLFVLSWAPYSAVALVAFAGYAHVLTPYMSSVPAVIAKASAIHNPIIYAITHPKYRVA   360
361 IAQHLPCLGVLLGVSRRHSRPYPSYRSTHRSTLTSHTSNLSWISIRRRQESLGSESEVGW   420
421 THMEAAAVWGAAQQANGRSLYGQGLEDLEAKAPPRPQGHEAETPGKTKGLIPSQDPRM     478
```

Figure S1. Sequence of isoform 1 of melanopsin protein (478 residues, 10 exons). Missense variants are depicted in yellow. Synonymous variants are depicted in green. Different exons are represented with alternate black and blue residues. Red residues are overlapping splicing sites. Figure was made by the authors using the sequences of OPN4 reported in www.ensemble.org(www.ensembl.org).
